# In-vitro validation of a new method to assess the clinical accuracy of complete arch impressions

**DOI:** 10.1007/s00784-025-06236-1

**Published:** 2025-02-25

**Authors:** Moritz Waldecker, Schessler Katherina, Bömicke Wolfgang, Rammelsberg Peter, Rues Stefan

**Affiliations:** 1 Department of Prosthetic Dentistry, Heidelberg University Hospital, University of Heidelberg, Heidelberg, Germany; 2 MZK 8.2 Poliklinik für Zahnärztliche Prothetik, Im Neuenheimer Feld 400, 69120 Heidelberg, Germany

**Keywords:** Digital impression, Intraoral scan, Dental impression technique, Dental impression materials, Reproducibility of results

## Abstract

**Objectives:**

To develop and validate a new method to acquire reference distances.

**Materials and methods:**

A method to accurately register the intraoral position of precision balls was developed to generate reference values for the in-vivo assessment of impression accuracy and tested in vitro. Therefore, metal occlusal veneers with a special abutment carrying precision balls were provisionally attached to specific positions on the patient’s dental arch (anatomical model with PMMA covered metal teeth). To register the precision ball positions, form-congruent counterparts were jointed to the abutments, adhesively fixed to a transfer aid, removed and digitized with a laboratory scanner. First, the distance determination using a transfer aid and a laboratory scanner was validated. Second, the process was tested for an anatomic situation.

**Results:**

When measuring distances using a transfer aid and a laboratory scanner, distances could be detected with an accuracy of less than 5 μm. Using the new test setup on the anatomical model, the intraoral scanners more accurately reproduced scan volumes up to one quadrant, with deviations between the actual scan data sets/plaster models and the reference data set of < 52 μm for Primescan, < 82 μm for Omnicam, and < 125 μm for conventional impression. Longer distances tended to be more accurately represented by the conventional impression (Primescan < 304 μm; Omnicam < 328 μm; conventional impression < 164 μm).

**Conclusions:**

The developed method seems suitable for determining the clinical accuracy of conventional and digital complete arch impressions.

**Clinical relevance:**

For determining the clinical accuracy of conventional or digital impressions, reference values are crucial.

## Introduction

Today, fixed dental prostheses are routinely fabricated using computer aided design/computer aided manufacturing. The starting point for computer aided design is to generate a three-dimensional (3D) data set of the intraoral situation. This can be done either by taking a conventional impression of the intraoral situation to produce a plaster model that is then digitized with a laboratory scanner or by digitizing the intraoral situation directly using an intraoral scanner. The use of intraoral scanners is increasing in dental practice. However, while for single-tooth crowns and short-span fixed partial dentures (FPD) fabricated on the basis of an intraoral scan, there are reports of a fit that is at least comparable and tends to be even better than on the basis of a CI [1], there are hardly any studies on the clinical accuracy of impressions or scans of more than half the jaw [2–8]. In in-vitro studies, the geometry of a reference models can be assessed with an accuracy exceeding that of the investigated dental scanning system, exemplarily by use of coordinate measurement machines. This is not possible in a clinical setting with the patient being the reference. Furthermore, if the evaluation should contain distances deviations and not only vertical surfaces deviations of each two aligned scans (for example an intraoral scan versus a laboratory plaster cast scan based on a conventional impression), it is necessary to define local reference points that can be reproduced with high accuracy. Reference points can be defined for any geometry such as a single tooth and transferred to a second scan [9], but it makes sense to use small and symmetric objects with known geometry such as precision balls with a few millimeters in diameter in this regard enabling reference point definition on a single scan basis [4, 5, 9–13]. Because of the above described problems, clinical accuracy studies often lack reference data and are therefore limited to determining deviations between different impression methods or evaluating some kind of repeatability, but not the deviation from the true clinical situation. A critical point with repeatability tests are systematic errors leading to very low deviations between each two scan repetitions although the scanning accuracy may have been poor. As can be seen from in-vitro tests with intraoral scanners, deviations from the reference follow, in general, a system-specific pattern [12].

Some attempts have been made to generate real reference data using reference bodies. These reference bodies include metal reference appliances that can be placed on the teeth [2], measuring gauges positioned in the palate [3], bars inserted transversely in the posterior palate [7], and precision balls fixed to the teeth [4–6]. There are, however, limitations to these approaches. Metal reference appliances can have negative optical properties [2], measuring gauges cover large areas of palatal teeth [3], and bars placed transversely in the posterior palate can only give information on this distance [7]. Kuhr et al. transferred and adhesively fixated precision balls in predefined positions to a patient’s dentition. With this methodology it is, however, not possible to place precision balls in the anterior region and it is difficult to use more than 4 balls [4]. To overcome these limitations, we sought to develop a new methodology. We also aimed to develop a reference that could be scanned both intraorally and extraorally. To address these goals, the aim of this study was to develop a new method to measure reference distances in vivo. Two null hypotheses were formulated. The first hypothesis was there is no difference between 2 different reference body designs when validating the test setup in vitro for determining reference distances using a transfer aid and a laboratory scanner. The second hypothesis was there is no difference between the intraoral scanners used or between the intraoral scanners and conventional impression when testing the method on an anatomical study model.

## Materials and methods

**Fig. 1:**
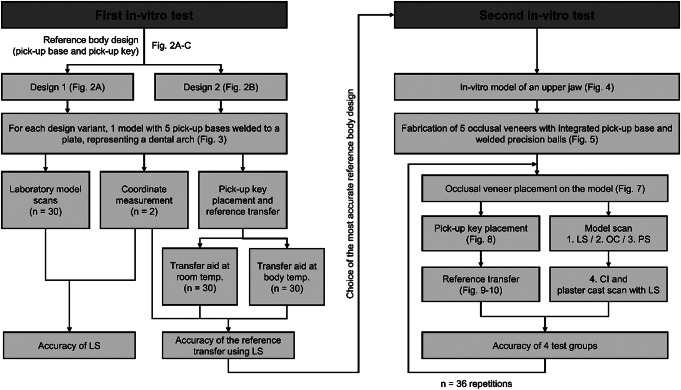
Study flowchart (LS, laboratory scanner; OC, Omnicam; PS, Primescan; CI, conventional impression)

To generate reference values to evaluate impression accuracy in future clinical trials, we aimed to develop and to test in vitro a method to accurately register the intraoral position of precision balls. The idea was to provisionally attach metal occlusal veneers with a special abutment carrying precision balls (pick-up bases, PBs) to specific positions on the patient’s dental arch. To register the precision ball positions, form-congruent counterparts (pick-up keys, PKs) leaving a small gap between precision ball and PK when jointed were used. Thus, all PKs could be individually placed on the respective PBs with a scannable impression material. After the impression material was cured, the PKs were adhesively fixed to a transfer aid and the transfer aid with the attached negatives (PKs + impression material) could be removed and digitized with a laboratory scanner. A study flowchart is shown in Fig. 1.

### First in-vitro test

#### Development of reference body designs

**Fig. 2:**
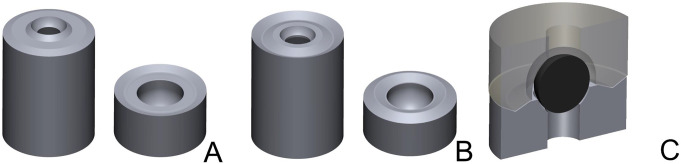
3D view of the reference body designs (left: pick-up base, right: pick-up key). **A**, Design variant (1) **B**, Design variant (2) **C**, Cross section through the pick-up base with precision ball and the jointed pick-up key for Design variant 2

Initially, 2 different reference body design variants were developed. For both variants, reference bodies consisted of 2 parts: an upper part, the PK, and a form-congruent lower part, the PB, which was initially connected to a cylinder of the same diameter and later to an occlusal veneer. All parts had rotational symmetry with respect to the vertical axis. The lower parts of both design variants were provided with a spherical mold into which a precision ball (nominal diameter = 3.175 mm; quality G3; shape deviation ≤ 0.08 μm, mean roughness value R_a_ ≤ 0.01 μm; variation of ball diameter ≤ 0.13 μm) was placed and welded at the basal opening of the spherical mold. The lower part of design variant 1 (D1) was divided into 3 surfaces in cross-sections from the outside to the inside: the first was a peripheral, inclined surface rising toward the center; the second was a horizontal plane; and the third was a central surface rising toward the precision ball (Fig. 2A). The lower part of design variant 2 (D2) consisted of a V-shaped notch along the outer circumference and a horizontal plane next to the precision ball (Fig. 2B). PKs were form-congruent counterparts, leaving a 0.5 mm wide gap along the precision ball surface (Fig. 2C).

#### Validation of the laboratory scanner

**Fig. 3:**
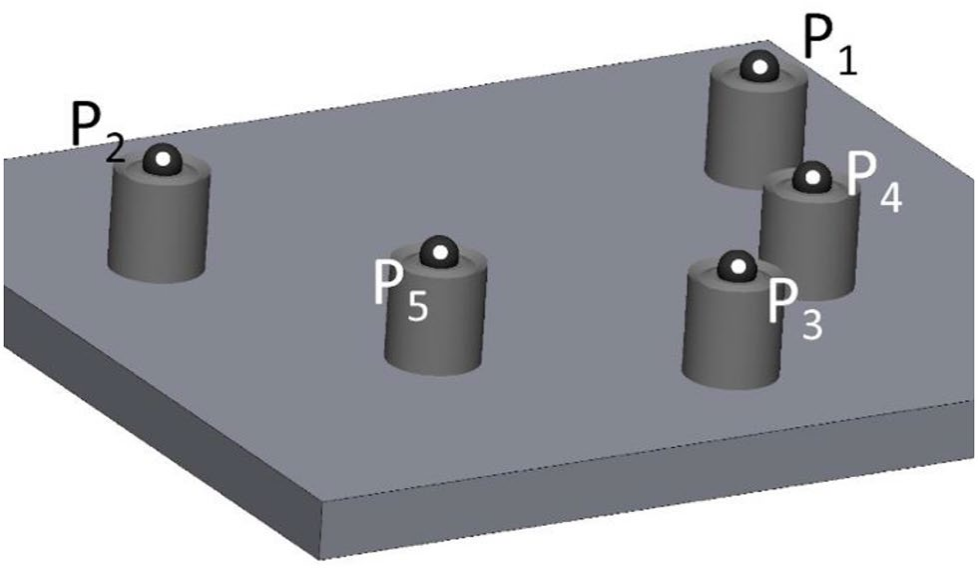
3D view of test model with reference body geometry D2 for validation of reference position determination using a transfer aid and a laboratory scanner. The pick-up bases were placed on an elliptical curve simulating a dental arch. P_1_ - P_5_ are the respective center points of the five precision balls

For both reference body design variants, a separate test model was produced. Both models were used to validate the use of a laboratory scanner (instead of a coordinate measurement machine) and later the transfer of the precision ball position with a transfer aid. Five cylinders with the respective lower part of the reference body and the corresponding pick-up keys were first designed with a reverse engineering software (Geomagic Design X, software version: 2020; 3D Systems, Moerfelden-Walldorf, Germany) and then milled from cobalt-chromium alloy blanks (Colado^®^ CAD CoCr4; Ivoclar Vivadent GmbH, Schaan, Liechtenstein). The 5 cylindrical PBs were welded elliptically, corresponding to the maxillary dental arch, onto a metal plate. To avoid reflections, the cylinders were abraded with 50-µm air borne alumina particles at 0.03 MPa. This low air pressure was sufficient for gloss removal and no noticeable geometry changes could be detected. To determine the spatial positioning of the precision balls on the cylinders and their respective center points, two repeat measurements were made with a coordinate measuring machine (MarVision 222; Mahr GmbH, Göttingen, Germany; accuracy < 1− 2 μm) at T_CM_ = 21 °C room temperature. This resulted in a point cloud consisting of 212–214 measuring points per precision ball. Ball centers were determined by minimizing the sum of all squared errors for any sphere with the given nominal radius and variable center position. In between the 2 repeat measurements made of each of the two models, the distances defined by each two respective precision ball centers deviated less than 1 μm from each other. The mean values of the precision ball center coordinates for each design variant were the respective reference values. These 5 center points were numbered as shown in Fig. 3, with P_1_ the position of the right second molar, P_2_ the position of the left second molar, P_3_ the position between the two central incisors, P_4_ the position of the right premolar, and P_5_ the position of the left premolar. For both models, reference distances were captured 30 times using a laboratory scanner (D2000; 3shape A/S, Kopenhagen, Denmark) with quality control software (Convince 2015; 3shape A/S, Kopenhagen, Denmark). The actual room temperature was recorded for each scan for later compensation of thermal expansion. The metal base was covered with a black cardboard to avoid reflections. For all scans, the positions of the centers of the 5 precision balls were determined by optimization (method of least squares; squared deviations at the triangle corner points were weighted with proportionate surface area using Matlab version R2022a; MathWorks, Natick, United States of America) and deviations between scan and reference distances defined by the centers of the precision balls were calculated. The coefficient of thermal expansion of the base plate (stainless steel) was α_T_ = 16∙10^− 6^K^− 1^. If the temperature during model scanning T_MS_ differed from the temperature during coordinate measurement, reference distances determined by model scanning (d_ref, MS_) were corrected as follows: d_ref_ = d_ref, MS_ / [1 + α_T_ ∙ (T_CM_ – T_MS_)]. These corrected values could be compared to the distances gained by coordinate measurement.

#### Validation of the reference distance determination using a transfer aid and a laboratory scanner

To use the position of the pick-up keys as a reference position for the (and in the future clinical) analysis, a stainless-steel transfer aid was fabricated. It had the same outline as the horizontal portion of a metallic rimlock tray (size XL) and a plate thickness of 3 mm. For both pick-up key designs, the transfer aid was validated at room temperature and in a preheated state to simulate the temperature change existing under clinical conditions when the transfer aid with attached PKs is removed from the patient´s mouth. First, the upper side of the 5 PKs and the underside of the transfer aid were abraded with 50-µm air borne alumina particles at 0.35 MPa, cleaned for 3 min in ethanol 70% in an ultrasonic bath, and then dried with oil-free air. A tray adhesive (Panasil^®^ Adhesive; Kettenbach GmbH & Co. KG, Eschenburg, Germany) for addition-curing silicones was applied to the inner surface of the PKs and air-dried for 5 min. The PKs were filled with a bite registration material based on polyvinyl siloxane (Futar^®^ Cut & Trim Fast; Kettenbach GmbH & Co. KG, Eschenburg, Germany) and placed on the PBs. The PKs possessed central openings on the upper side allowing excess material to flow out. After curing, silicone excess on the top of the pick-up keys was removed and these were adhesively bonded to the transfer aid using an autopolymerizing adhesive resin cement (Panavia 21; Kuraray Europe GmbH, Hattersheim am Main, Germany).

For tests with the transfer aid at room temperature, 30 min time were needed to ensure complete curing of the adhesive. The exact room temperature T_AF_ during adhesive fixation was recorded. The transfer aid was first fixed for 1 min and then held in situ for 29 min without pressure. After removal, the transfer aid was disinfected (PrintoSept-ID, Alpro Medical GmbH, St. Georgen, Germany) for 5 min according to the clinical procedure. After at least 30 min, the transfer aid with the pick-up keys showing the precision ball impressions was digitized using a laboratory scanner (D2000; 3shape A/S, Kopenhagen, Denmark) with quality control software (Convince 2014; 3shape A/S, Kopenhagen, Denmark) to generate a digital data set in standard tessellation language (STL) file format with a rather uniform triangle edge length of 50 μm. For all scans, the positions of the five precision ball centers were determined by optimization (method of least squares; squared deviations at the triangle corner points were weighted with proportionate surface areas associated with the corner points using Matlab, software version: R2022a; MathWorks, Natick, United States of America) and deviations between scan and reference distances defined by the precision ball centers were calculated.

The procedure for the test with the preheated transfer aid differed from that at room temperature in that the transfer aid was stored in an incubator for 60 min at 55 °C after pretreatment before it was then bonded to the PKs. The preheated transfer aid (surfaces not relevant for bonding were insulated to enable slow cooling) was removed from the oven and bonded to the pick-up keys. The transfer aid was fixed for 1 min and then held in situ for 9 min without pressure. With 55 °C starting temperature, the transfer aid had a temperature of 32.5 °C after 5 min. At this time, it was not possible any more to manually remove the PKs from the transfer aid and it was assumed that reference positions of the PKs refered to this temperature. The aim was for the transfer aid to have a temperature of 32.5 °C since this corresponded to the mean temperature that the transfer aid had assumed during a preliminary test with an intraoral setting time of 10 min.

Again, for both experiments (transfer aid at room or body temperature), the transfer aid with the PKs was scanned (D2000; 3shape A/S, Kopenhagen, Denmark) and the room temperature during each scan process noted. Due to temperature differences between the registration scan (T_RS_) and the temperature during adhesive fixation (T_AF_), the reference distances based on the registration scan (d_ref, RS_) had to be corrected by the thermal expansion of the stainless steel transfer aid: d_ref_ = d_ref, RS_ / [1 + α_T_ ∙ (T_RS_ – T_AF_)].

### Second in-vitro test

#### Process testing for anatomic situations

**Fig. 4:**
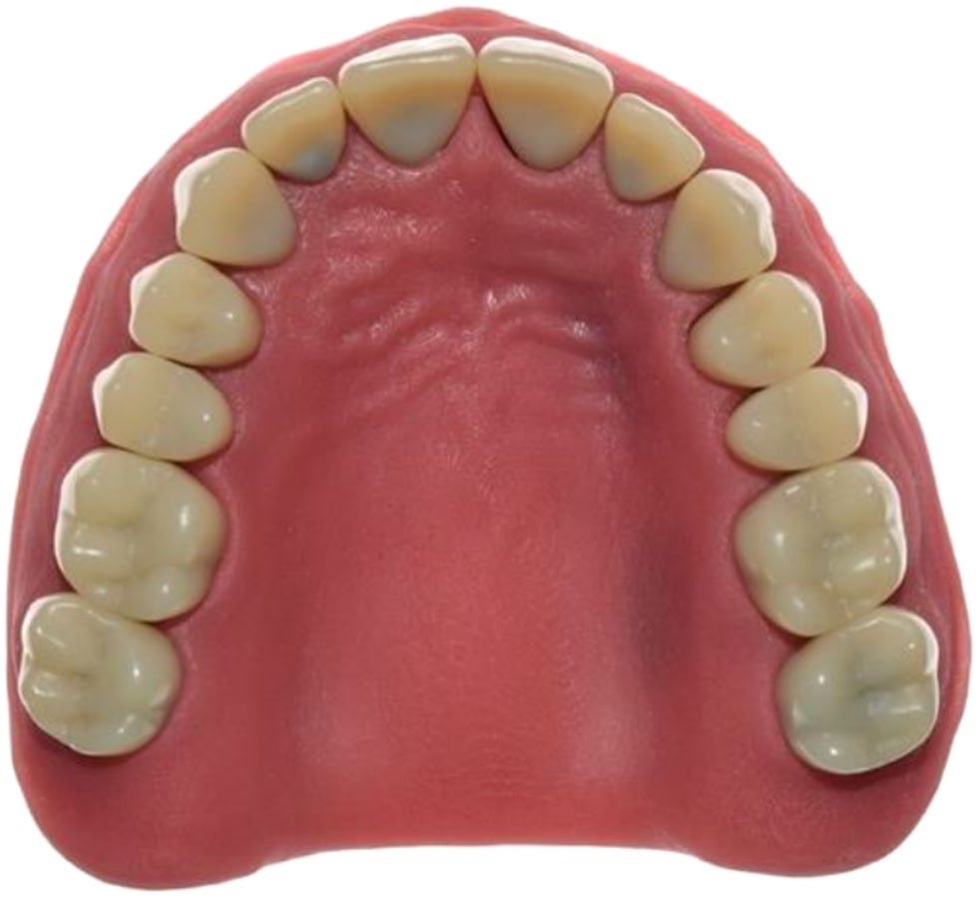
Occlusal view of study model of fully dentate maxilla model

**Fig. 5:**
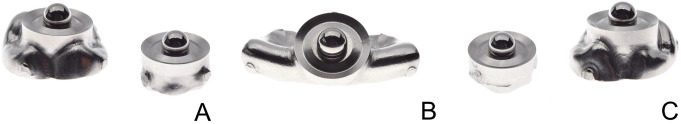
Occlusal veneers to detect dimensional changes in digital and conventional impressions. Images were taken before moderate sandblasting which was necessary for use of intraoral scanners (gloss removal). **A**, Occlusal veneer for left first premolar and left second molar. **B**, Occlusal veneer for central incisors. **C**, Occlusal veneer for right first premolar and right second molar

**Fig. 6:**
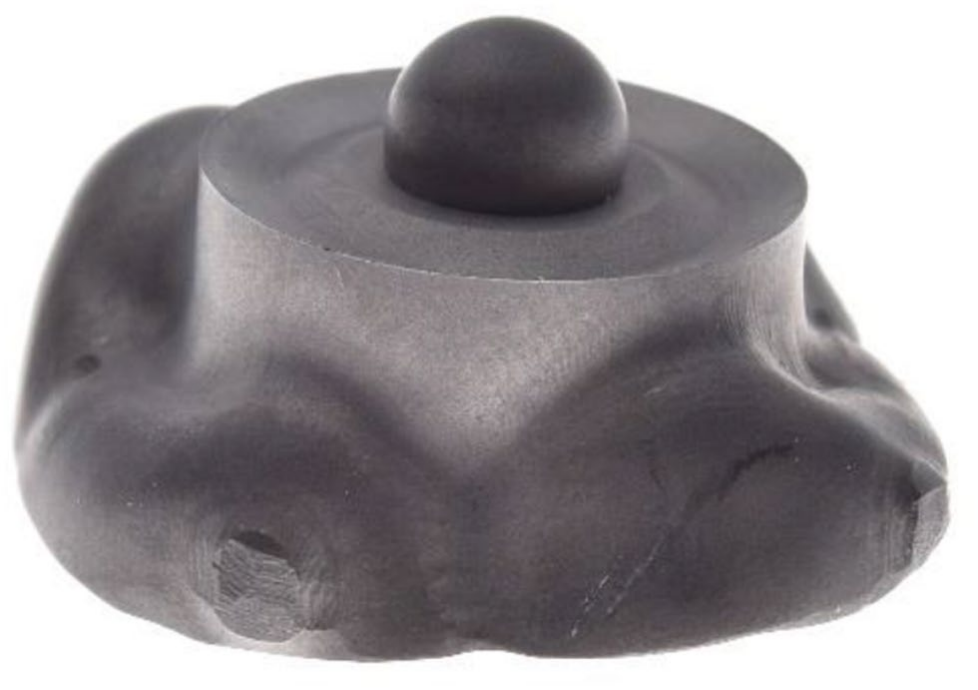
Sandblasted occlusal veneer for left second molar

**Fig. 7:**
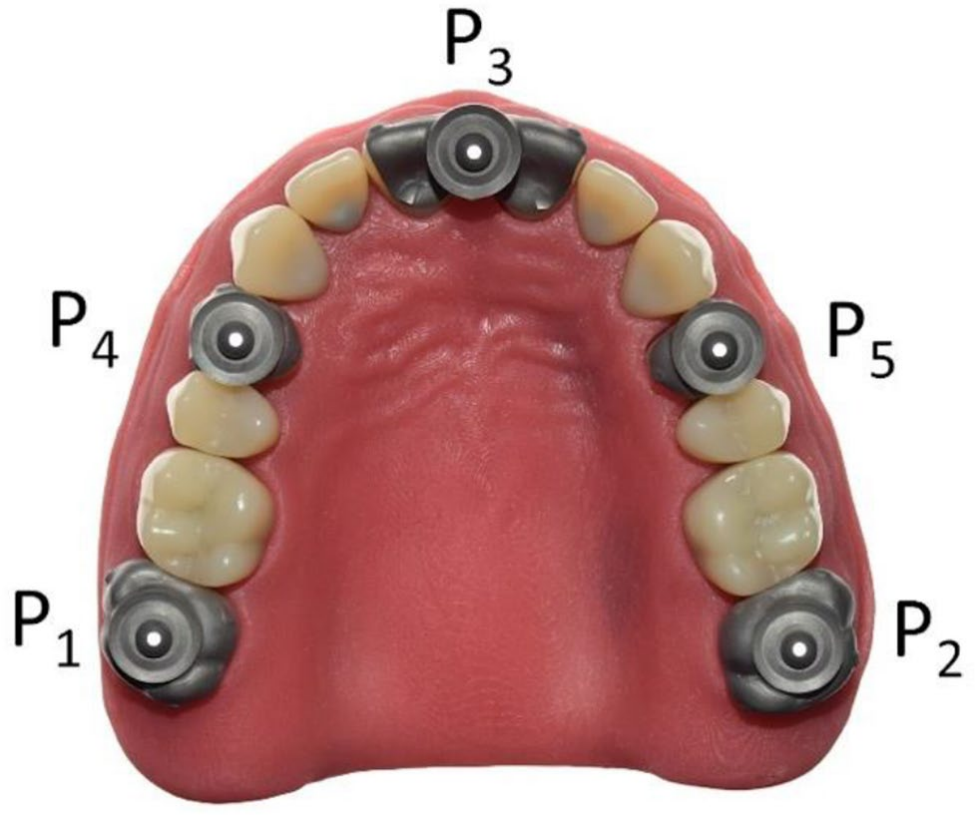
Occlusal view of study model with sandblasted occlusal veneers fixed on both second molars, first premolars, and central incisors

**Fig. 8:**
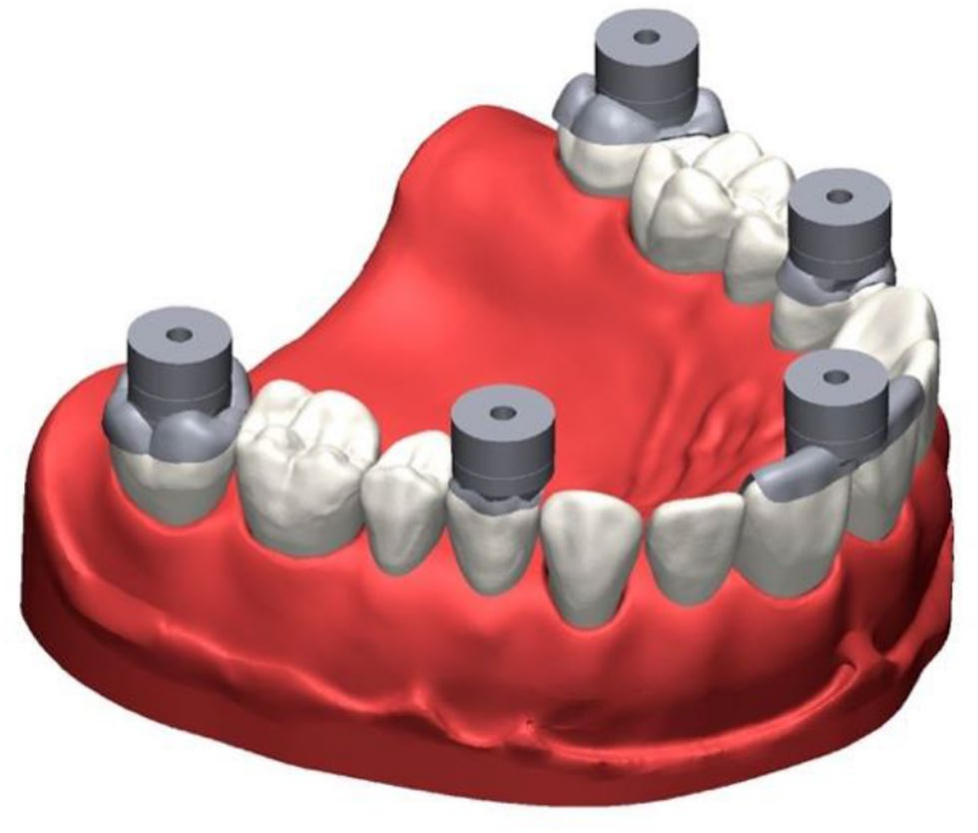
3D view of pick-up keys placed on pick-up base

**Fig. 9:**
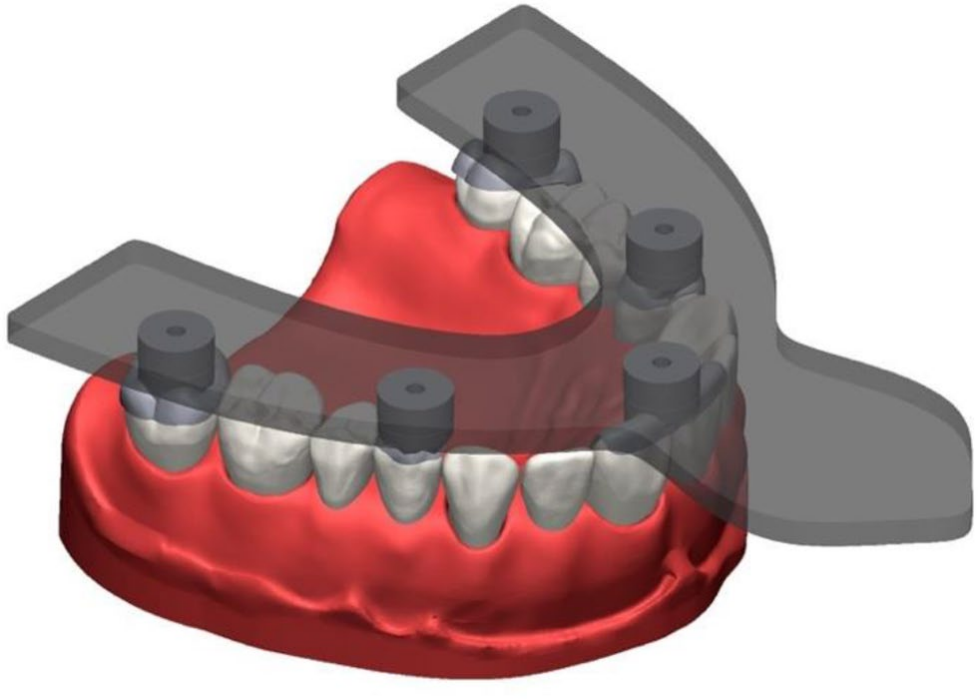
3D view of transfer aid with adhesively attached to pick-up keys

**Fig. 10:**
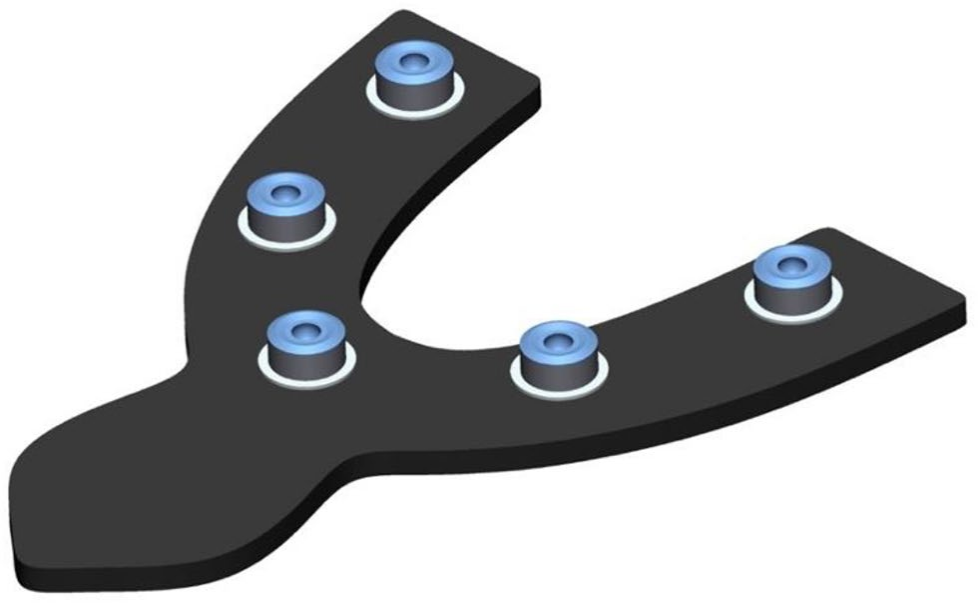
3D view of transfer aid with adhesively attached pick-up keys including the cured scannable impression material for detecting precision ball center positions

For process testing for anatomic situations, a test model was produced simulating a fully dentate maxilla with 14 teeth (Fig. 4). The teeth were fabricated in two layers and consisted of a cobalt-chromium alloy core (Remanium^®^ star MD II; Dentaurum GmbH & Co. KG, Ispringen, Germany) to which a polymethylmethacrylate crown (Ivotion Dent A3; Ivoclar Vivadent GmbH, Schaan, Liechtenstein) in the desired tooth shape was adhesively attached. The teeth were welded to a steel base, which represented the maxilla. The metal base was covered with a stereolithographically produced gingival mask (FREEPRINT^®^ gingiva; Detax GmbH & Co. KG, Ettlingen, Germany). Occlusal veneers were designed with a reverse engineering software (Geomagic Design X; 3D Systems, Moerfelden-Walldorf, Germany) for the second molars, first premolars, and first incisors. The incisal veneers were blocked together. Each occlusal veneer served as the basis for a pick-up base with the design 2 (Fig. 5A-C). This design was based on the results of the first in-vitro test. During the CAD process, care was taken to ensure that all 5 PB geometries were arranged in one plane to allow gap-free bonding with the transfer aid later on. The 5 occlusal veneers with their pick-up base were milled from cobalt-chromium alloy blanks (Colado^®^ CAD CoCr4; Ivoclar Vivadent GmbH, Schaan, Liechtenstein) in one piece. The following procedure was performed 36 times. Each time, the 5 occlusal veneers were completely removed, composite residues were removed by abrasion with 50-µm air borne alumina particles at 0.35 MPa (Fig. 6), and the 5 occlusal veneers were fixed again to the teeth with a flowable composite (Rebilda DC; Voco GmbH, Cuxhaven, Germany) (Fig. 7). After the occlusal veneers were fixed, the test model was digitized using a laboratory scanner (LS, D2000; 3shape A/S, Kopenhagen, Denmark) with quality control software (Convince 2014; 3shape A/S, Kopenhagen, Denmark) to generate a digital control data set in STL file format. Next, the model was inserted in a phantom head, which was fixed on a dental chair in a windowless room with artificial lighting to simulate clinical conditions while scans and conventional impressions (CI) were taken. The room temperature was 23.9 ± 0.3 °C and the relative humidity was 32.5 ± 10.7% for all scans. All impressions were performed by a calibrated investigator with experience in scanning (S.K.). The intraoral scanners used were Omnicam AC (OC) (Dentsply Sirona, Charlotte, United States of America) and Primescan (PS) (Dentsply Sirona, Charlotte, United States of America). The order of the scanners was: Omnicam first, Primescan second. Both were calibrated with a device-specific calibration aid before use. The scans were performed using the scanning strategy recommended by the manufacturer (Table 1) and were postprocessed using manufacturers’ software (Sirona Connect v5.2; Dentsply Sirona, Charlotte, United States of America). Scans were exported in STL file format for further evaluation. The position of the pick-up keys was registered with the transfer aid as described above (Figs. 8, 9 and 10). The CI was performed using a dual phase impression technique with polyvinyl siloxane (PVS) material (Aquasil Ultra + XLV and Aquasil Ultra Plus Heavy, Dentsply Sirona, Charlotte, United States of America). All CIs were removed from the model after 15 min, which is 3 times the clinical setting time for PVS. The extended setting time at room temperature was necessary because shrinkage effects were observed 10 min after mixing in pretests. The metallic rimlock trays were individualized with a palatal stop and a dorsal dam to ensure a permanent distance between the tray and the teeth and positional stability during the setting time, and to support a seamless flow of the impression material to the tooth row. CIs were disinfected for 5 min (PrintoSept-ID, Alpro Medical GmbH, St. Georgen, Germany), rinsed with water, air-dried with oil-free air, and then poured with type IV gypsum (esthetic-base gold, dentona AG, Dortmund, Germany) no earlier than 30 min after disinfection. The plaster casts were digitized using a laboratory scanner (D2000, 3shape A/S, Kopenhagen, Denmark) with quality control software (Convince 2015, 3shape A/S, Kopenhagen, Denmark) to generate a digital data set in STL file format. For all scans, the positions of the 5 precision ball centers were determined by optimization (method of least squares; squared deviations at the triangle corner points were weighted with proportionate surface area using Matlab, software version: R2022a, MathWorks, Natick, United States of America) and deviations between scan and reference distances defined by the centers of the precision balls were calculated. The center points were numbered according to the system used for the test models with P_1_ on the right second molar, P_2_ on the left second molar, P_3_ between the two central incisors, P_4_ on the right premolar, and P_5_ on the left premolar.

**Table 1:** Specifications for intraoral scanners

Intraoral scanner	Software	Scan sequence	Hardware settings
Omnicam	Sirona Connect v5.2	First scan quadrant: P (45° and 90°), O, B (45° and 90°) Second scan quadrant: P (90° and 45°), B (45° and 90°), O	CPU, Intel^®^ Core™ i7-5820 K 3.30 GHz RAM, 32.0 GB Display card, AMD Radeon™ RX 470 Series
Primescan	Sirona Connect v5.2	P, O, B	CPU, Intel^®^ Core™ i7-8700 3.20 GHz RAM, 32.0 GB Display card, AMD Radeon RX 570 Series

B, buccal; O, occlusal; P, palatal

For the first in-vitro test, data were not normally distributed, so a Mann-Whitney-U test was performed to determine whether the difference in means of the variable of interest between the two designs was significant at the 5% level in each subgroup. For the second in-vitro test, a Kruskal-Wallis one-way analysis of variance was performed to determine whether the difference in means of the variables of interest between both intraoral scanners and the CI was significant at the 5% level. If the overall test was statistically significant, pairwise comparisons were performed (Mann-Whitney-U test).

## Results

### First in-vitro test

#### Validation of the laboratory scanner

**Fig. 11:**
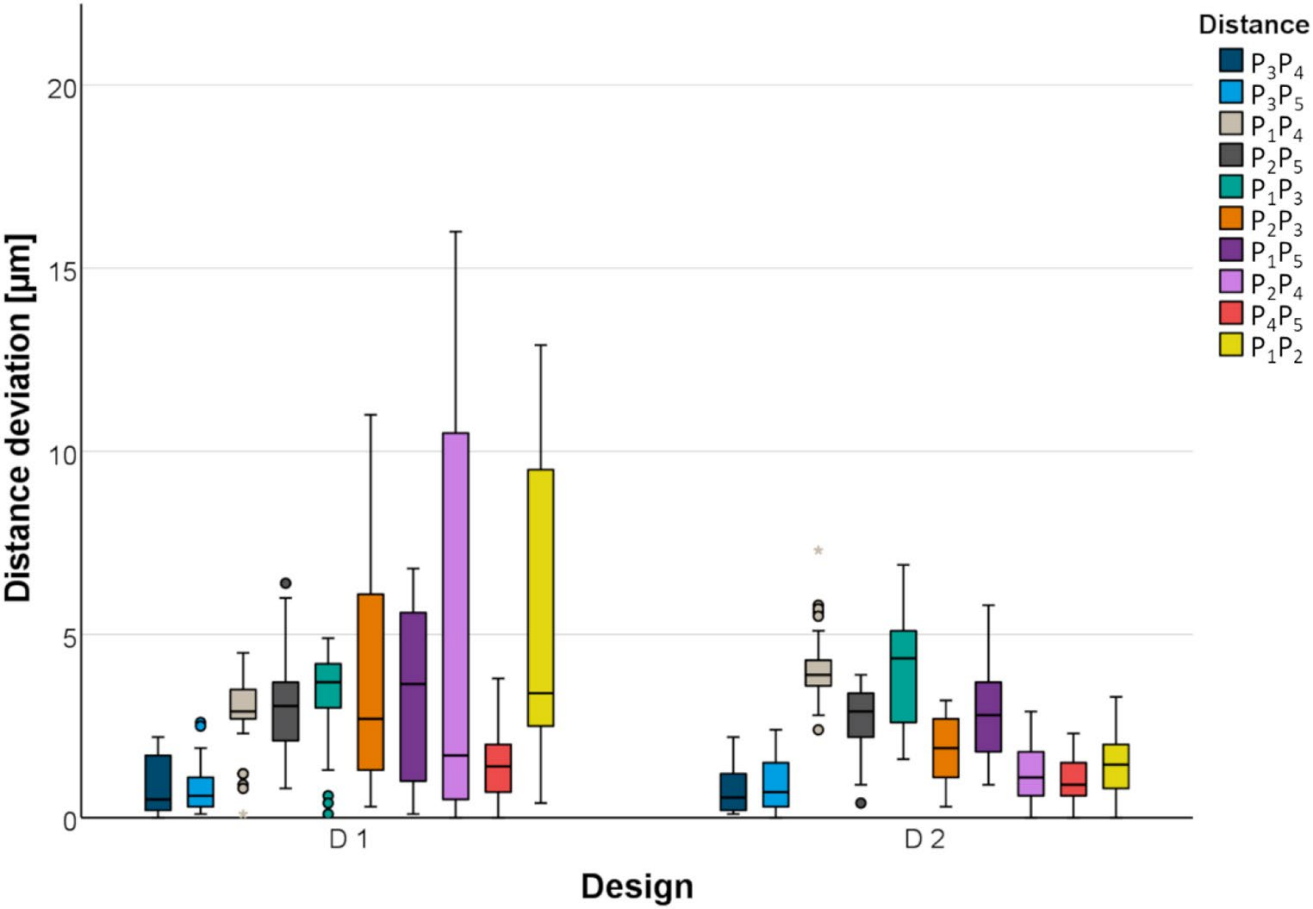
Absolute distance deviations displayed for test models with D1 and D2 occlusal veneer designs scanned by laboratory scanner

The absolute distance deviations for the two test models with D1 and D2 occlusal veneer designs scanned by the laboratory scanner are presented in Fig. 11. Overall, the test model with the D2 occlusal veneer design caused smaller distance deviations (absolute mean distance deviation: 2.9 ± 2.9 μm for D1 and 2.1 ± 1.6 μm for D2; absolute maximum deviations: 16 μm for D1 and 7.3 μm for D2). There were no statistically significant differences between the two reference body designs in most cases. There was a significant difference with an advantage for D2 for distances P_1_P_2_ and P_2_P_3_ (*P* <.031). For distance P_1_P_4_, there was a significant difference with an advantage for D1 (*P* <.001).

#### Validation of the reference distance determination using a transfer aid and a laboratory scanner

**Fig. 12:**
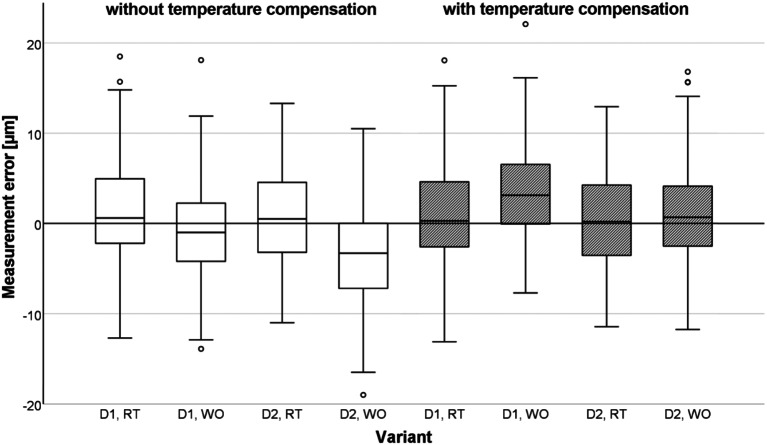
Measurement error of transfer aid displayed for occlusal veneer designs D1 and D2 at room temperature (RT) and preheated in warming oven (WO) with and without temperature compensation

Figure 12 shows the relative measurement error of the transfer aid for the D1 and D2 occlusal veneer designs at room temperature (RT) and preheated in a warming oven (WO) with and without temperature compensation. The absolute mean measurement error without temperature compensation was 4.3 ± 3.5 μm for D1 and 4.3 ± 3.1 μm for D2 at RT, and 4.0 ± 3.1 μm for D1 and 5.1 ± 3.9 μm for D2 preheated in a WO. The absolute mean measurement error with temperature compensation was 4.3 ± 3.4 μm for D1 and 4.3 ± 3.0 μm for D2 at RT, and 4.7 ± 3.7 μm for D1 and 4.1 ± 3.2 μm for D2 preheated in a WO. The absolute maximum error for all groups was between 13 and 19 μm. The absolute mean measurement error at room temperature was slightly but not significantly smaller for D1 than for D2. Preheating the transfer aid in a warming oven reduced the mean absolute measurement error for D2, so the difference in distances P_1_P_5_ and P_3_P_4_ was statistically significant (*P* <.018).

### Second in-vitro test

#### Process testing for anatomic situations

**Fig. 13:**
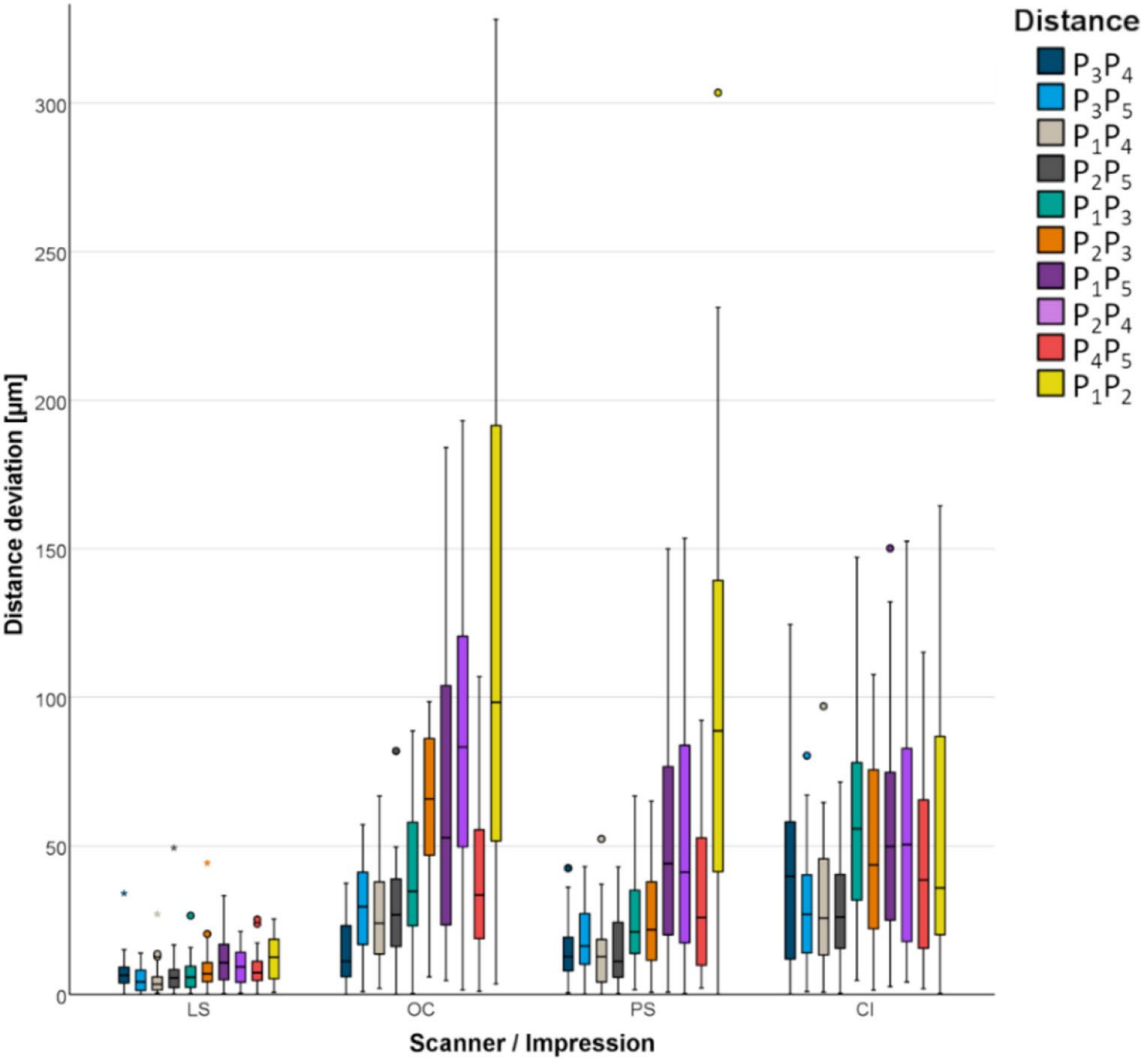
Absolute distance deviations in relation to transfer aid displayed for all test groups (LS, laboratory scanner; OC, Omnicam; PS, Primescan; CI, conventional impression)

Figure 13; Table 2 show the absolute distance deviations for all test groups when process testing for anatomic situations. The control scans with LS showed a mean deviation from the reference of < 13 μm, which is in the range of twice the scanning accuracy of the used LS. The maximum deviation was 49 μm. Short distances (P_3_P_4_, P_3_P_5_, P_1_P_4_, and P_2_P_5_) and medium distances (P_1_P_3_ and P_2_P_3_) were represented more accurately by the digital impression. For short distances (corresponding to a situation of an FPD with up to 4 units), the deviations were a maximum of 52 μm for PS, 82 μm for OC and 125 μm for CI. For medium distances (corresponding to a quadrant scan), the deviations were a maximum of 67 μm for PS, 98 μm for OC and 147 μm for CI. The anterior segment (distance P_4_P_5_) was depicted most accurately by PS (up to 92 μm), followed by OC (up to 107 μm), and the CI (up to 115 μm). In addition, deviations increased over the anterior segment. Regardless of the scanning system and the impression technique, the first sextant (distance P_1_P_5_) was represented more accurately than the second sextant (distance P_2_P_4_). Sextants were accurately represented when using both the CI and PS with deviations up to 154 μm compared with deviations up to 193 μm with OC. Regardless of the scanning system and impression technique, the greatest deviations were found over the cross-arch distance (P_1_P_2_). Deviations were higher when using OC (up to 328 μm) and PS (up to 304 μm) than when using CI (up to 164 μm). The differences were statistically significant for all distances (*P* <.007) except P_2_P_5_ and P_4_P_5_ (*P* >.394). When comparing the two scanning systems, distance deviations were significantly lower with PS for all distances (*P* <.004) except P_1_P_2_ and P_3_P_4_ (*P* >.321). The CI showed a significant advantage over PS for the distance P_1_P_2_ (*P* =.005). For all other comparisons, except P_2_P_4_ (*P* =.618), PS was significantly more accurate than CI (*P* <.025). The comparison of OC with CI yielded a less clear result. There were significantly lower deviations for distances P_1_P_3_ and P_3_P_4_ with OC (*P* <.044), whereas significantly lower deviations were measured for distances P_1_P_2_, P_2_P_3_, and P_2_P_4_ with the CI (*P* <.048).

**Table 2:** Absolute distance deviations in relation to transfer aid

Group	Distance	Mean	SD	Minimum	Median	Maximum
		[µm]				
LS	P_3_P_4_	7	6	< 1	6	34
	P_3_P_5_	5	4	< 1	4	14
	P_1_P_4_	5	5	< 1	3	27
	P_2_P_5_	7	8	< 1	6	49
	P_1_P_3_	7	5	< 1	6	27
	P_2_P_3_	9	8	< 1	7	44
	P_1_P_5_	11	8	< 1	11	33
	P_2_P_4_	9	6	< 1	9	21
	P_4_P_5_	9	6	< 1	7	25
	P_1_P_2_	12	8	< 1	13	26
OC	P_3_P_4_	14	11	< 1	11	37
	P_3_P_5_	29	15	< 1	30	57
	P_1_P_4_	26	16	2	24	67
	P_2_P_5_	28	16	< 1	27	82
	P_1_P_3_	41	25	< 1	35	89
	P_2_P_3_	63	27	6	66	99
	P_1_P_5_	67	54	5	53	184
	P_2_P_4_	85	51	1	83	193
	P_4_P_5_	39	28	1	33	107
	P_1_P_2_	125	94	4	98	328
PS	P_3_P_4_	15	11	1	13	43
	P_3_P_5_	18	11	< 1	16	43
	P_1_P_4_	13	11	< 1	13	52
	P_2_P_5_	14	12	< 1	11	43
	P_1_P_3_	24	17	2	21	67
	P_2_P_3_	27	18	1	22	65
	P_1_P_5_	53	40	1	44	150
	P_2_P_4_	51	40	< 1	41	154
	P_4_P_5_	33	27	2	26	92
	P_1_P_2_	98	69	< 1	89	304
CI	P_3_P_4_	39	30	< 1	40	125
	P_3_P_5_	30	21	1	27	80
	P_1_P_4_	30	22	1	26	97
	P_2_P_5_	29	18	< 1	26	72
	P_1_P_3_	59	37	5	56	147
	P_2_P_3_	48	32	1	44	108
	P_1_P_5_	55	37	3	50	150
	P_2_P_4_	55	40	4	50	153
	P_4_P_5_	43	32	2	39	115
	P_1_P_2_	55	46	< 1	36	164

CI, conventional impression; LS, laboratory scanner; OC, Omnicam; PS, Primescan; SD, standard deviation

## Discussion

The null hypotheses that there is no difference between 2 different reference body designs, and that there is no difference either between the intraoral scanners used, or between the intraoral scanners and the CI had to be rejected.

The basic idea of determining the accuracy of conventional or digital impressions in vivo using geometric reference bodies has already been pursued. As mentioned above, the reference bodies used here, and their intraoral arrangement have certain specific limitations. The present study has in common with these studies is that by defining distances, concrete statements can be made about the deviations in size and location in the dental arch. This appears to be particularly advantageous compared to the generally often chosen evaluation strategy using a best-fit alignment, in which only deviations between two surface data sets can be specified.

A unique feature of the new transfer aid is the ability to pick up reference positions at any point in the dental arch regardless of whether it is the mandible or maxilla. This flexibility allows a distribution over the dental arch, making it possible to determine scanning accuracy in the anterior segment and to examine jaws with different partial dentitions in the future.

The most critical point in clinical accuracy studies is generating a reference data set. With the aim of developing a reference that could be scanned both intraorally and extraorally, the registration and subsequent digitization of the precision ball positions was the most critical point. With a mean measurement error of < 5 μm, the registration method can be considered adequate for reference generation. An advantage of the new method, particularly in clinical studies, is that individual patient-related reference distances can be determined by custom laboratory scans of the transfer aid. This approach saves the preparation time needed when using a coordinate measurement machine. Furthermore, tactile measurement of resilient surfaces (the PVS layer) is critical.

Two different reference body designs were tested in this study. The results show that the reference body geometry can influence the scanning accuracy. Absolute maximum distance deviations between 2 precision balls fixed on reference bodies were twice as large with design 1 as with design 2. Consequently, only those reference bodies with the least influence on scanning accuracy should be used when assessing scanning accuracy.

Another advantage of these reference bodies is that they do not disadvantage the conventional impression due to deformation, like a bar crossing the palate can do [7]. However, the height of the reference bodies reduces the impression accuracy of the conventional impression, which is why care was taken to minimize the height during the design process. In addition, the arrangement of the reference bodies favors the scanning accuracy of the IOS in that the reference body on the central incisors introduces an additional landmark to the anterior region, which is otherwise generally poor in landmarks.

The presented approach of designing reference bodies individually for each patient is time-consuming and costly compared to other methods that use ready-made reference bodies such as precision balls or a bar. However, the individual reference bodies are not fixed in terms of their position in the dental arch based on geometric dimensions or the planned position with a transfer aid.

Various factors influence the scanning accuracy [14]. To achieve the best possible scanning results, the latest software version of the intraoral scanner was used. Because the scanning strategy can influence scanning accuracy, the scanning strategies recommended by the manufacturer for the two scanning systems were used when performing the scans [15–19]. In addition, to avoid any influence of the examiner, all scans were performed by a calibrated examiner with experience in scanning [20].

Scanners in general have problems capturing metal or very shiny surfaces. Therefore, to avoid reflections, the occlusal veneers were abraded with 50-µm air borne alumina particles at 0.03 MPa. This low air pressure was sufficient for gloss removal and no noticeable geometry changes could be detected. As the deviations from the reference measurement were < 5 μm, it can be stated that the matt metal surfaces had no influence on the scanning accuracy.

Intraoral scanners should be calibrated regularly [21]. The intraoral scanners used were therefore calibrated with their respective calibration aid before each run. However, even with correct calibration, identical scanning systems from the same manufacturer can still show differences in scanning accuracy [10]. Furthermore, the calibration set supplied by the manufacturer does not necessarily lead to the best possible scan results [10]. This effect is not clinically relevant for scans up to one quadrant, but does influence scans beyond one quadrant [10]. The results of this study must therefore be viewed critically and should only be used as a guide for interpretation, as it cannot be assumed that the best possible scanner/calibration aid combination was used.

At the same time, the use of a fully dentate model with restoration-free teeth represents a best-case scenario. Edentulous ridge sections can reduce scanning accuracy [12, 22]. The extent of this influence appears to depend on the distribution of the remaining teeth and the measured variable [12, 22]. Waldecker et al. found no clear difference in horizontal and vertical distances between a partially edentulous and fully dentate maxilla. However, partially edentulous dentition significantly influenced the alignment of the individual distances, resulting in significantly wider dental arches compared to the reference [12].

The mean and maximum absolute distance deviations measured in this study are similar to those reported in other studies using study models with identical dimensions, so can therefore be considered realistic [9, 10, 22]. A comparison of the absolute values with other studies is difficult, as distance deviations depend on the scan path length [23]. However, this is generally not specified in most studies. Furthermore, the evaluation strategies (best-fit vs. distance deviations) are different, lead to different results and are therefore not comparable.

## Conclusions

It can be concluded that the newly developed and in vitro tested method is suitable for registering reference positions. The presented method can determine deviations < 5 μm. The clinical applicability of the method has yet to be verified.

## Data Availability

No datasets were generated or analysed during the current study.
