# Correction to: In-vitro validation of a new method to assess the clinical accuracy of complete arch impressions

**DOI:** 10.1007/s00784-025-06292-7

**Published:** 2025-03-29

**Authors:** Moritz Waldecker, Katherina Schessler, Wolfgang Bömicke, Peter Rammelsberg, Stefan Rues

**Affiliations:** 1 Department of Prosthetic Dentistry, Heidelberg University Hospital, University of Heidelberg, Heidelberg, Germany; 2 MZK 8.2 Poliklinik für Zahnärztliche Prothetik, Im Neuenheimer Feld 400, 69120 Heidelberg, Germany

**Correction to: Clinical Oral Investigations**

10.1007/s00784-025-06236-1

The order of the names and surnames of the co-authors have been reversed.

The author’s names should be as follows:

Name: Katherina

Surname: Schessler

Name: Wolfgang

Surname: Bömicke

Name: Peter

Surname: Rammelsberg

Name: Stefan

Surname: Rues

The original article has been corrected.

